# Patient-related characteristics or local tradition: what predicts the admission to a locked ward or the use of coercive measures in psychiatric inpatient treatment?

**DOI:** 10.1007/s00406-024-01936-3

**Published:** 2024-11-12

**Authors:** Lisa Hochstrasser, Daniela Fröhlich, Julian Moeller, Andres R. Schneeberger, Stefan Borgwardt, Undine E. Lang, Christian G. Huber

**Affiliations:** 1 Universität zu Lübeck, Ratzeburger Allee 160, 23562 Lübeck, Germany; 2 Universitäre Psychiatrische Kliniken Basel, Universität Basel, Wilhelm Klein-Str. 27, Basel, 4002 Switzerland; 3 Zürcher Hochschule für angewandte Wissenschaften, Katharina-Sulzer-Platz 9, Winterthur, 8400 Switzerland; 4 Fakultät für Psychologie, Universität Basel, Missionsstrasse 60/62, Basel, 4055 Switzerland; 5 Health Psychiatry, University of California, 8950 Villa La Jolla Drive, San Diego, 92037 USA; 6 Department of Psychiatry and Behavioral Sciences, Albert Einstein College of Medicine, 3331 Bainbridge Avenue, Bronx, NY 10467 USA; 7 Institute of General Practice and Interprofessional Care, University Hospital Tuebingen, Tuebingen, Germany

**Keywords:** Acute psychiatric treatment, Aggression, Coercive treatment, Closed ward, Compulsory treatment, Predictor

## Abstract

Prior research shows that locked doors and coercive measures are not only applied due to safety concerns, but also due to the specific local tradition of an institution. We examined the association of the use of coercive measures and the admission to a locked ward with person-related characteristics compared to the admission to a specific clinic. In this 15-year, naturalistic observational study, we examined 230,684 admissions to 14 German psychiatric inpatient clinics from Jan 1, 1998, to Dec 31, 2012. To analyze the degree to which admission to a locked ward and coercive measures (received vs. not received) were connected with person- and clinic-specific factors, two-step logistic regression analyses were applied. 27% of the variance of the admission to a locked ward were explained by person-related characteristics (Nagelkerke r^2^ = 0.269). By adding the clinic the person was admitted to, the explained variance increased by 15% (Nagelkerke r^2^ = 0.418). 36% of the variance of the use of coercive measures were explained by person-related characteristics (Nagelkerke r^2^ = 0.364). By adding the clinic the person was admitted to, the explained variance increased by 4% (Nagelkerke r^2^ = 0.400). The local tradition of a psychiatric clinic seems to play a more prominent role for the decision to admit a person to a locked ward than for the decision to use coercive measures. Clinicians should be made aware of the connection of local traditions with clinical pathways in acute psychiatry to avoid unnecessary admissions to locked wards.

## Introduction

The treatment on locked wards and the use of coercive measures are still very common in mental health care settings [1–4]. Following the medico-ethical guidelines of the Swiss Academy of Medical Sciences, the application of a coercive measure is indicated in cases where a risk of harm to the patient or others cannot otherwise be averted [5]. International guidelines state that coercive measures should only be used when all the other less restrictive approaches failed [6]. The constraints to personal freedom these treatment settings impose is ethically problematic, and is acceptable from an ethical point of view only under certain conditions: The least restrictive alternative has to be used and its duration has to be kept to a minimum, the patient’s rights have to be granted, a patient’s relatives or guardians should be informed and the procedure has to follow established national and local protocols [7–12].

Clearly, there are certain situations, in which these measures are needed to protect patients from endangering themselves or others [13, 14]. On the other hand, the intended benefits of locked wards may not always be achieved [15, 16]. Furthermore, there are several risks and disadvantages of coercive measures and locked doors in psychiatric inpatient treatment [17, 18]. One of the most relevant disadvantages is the negative impact on the therapeutic relationship with the patients receiving coercive measures and the ward atmosphere in general [19, 20]. Furthermore, patients’ satisfaction with treatment and care may be lower on locked than on open wards [21] and is reduced by other coercive measures as involuntary admission or perceived coercion during the current admission [22]. In addition, there is a trend towards reduced compliance after involuntary hospitalization [23]. In a systematic review Chieze [24] even found a correlation between seclusion and restraint and the development of post-traumatic stress disorder. Considering the sometimes dubious benefits and the numerous disadvantages of coercion in psychiatric treatment, it is obvious that the decision to admit a patient to a locked ward or to use coercive measures should be made with great care after thorough deliberation of the relevant patient-related characteristics.

However, it is unclear, on which basis the decision for the admission to a locked ward or the use of coercive measures is made in clinical reality. On one hand certain patient characteristics appear to influence the decision-making process regarding the use of coercive measures: Patients with lower age and male gender have a greater risk to experience mechanical restraint, seclusion and compulsory medication [25, 26]. Furthermore, patients with a diagnosis of schizophrenia [27] and psychotic episodes in general [28] are more likely to get in touch with coercive measures. A higher severity of symptoms at the time of admission led to a higher risk for seclusion [29, 30]. On the other hand there seem to be further impact factors on the use of coercion in psychiatric inpatient settings in addition to the official guidelines or – to put it in another way – the clinician-based individual evaluation of the need for admission to a locked ward and coercive measures might be influenced by other factors, which need to be investigated. Regarding the admission to locked wards, Rittmannsberger [31] found that locking policies are determined by local tradition to a large extent. Huge differences between countries, hospitals and wards were found. A review article of Luciano et al. [32] about the prediction of using coercive measures in mental health care settings found that coercive measures were predicted by patients’ clinical and sociodemographic features, staff characteristics and ward-related factors. Korkeila [33] also found a “facility effect” regarding the application of coercive measures: The choice between use of either seclusion or restraint and the pattern of using them (type of coercive measure, frequency, and duration) differed according to the center. Husum [34] found a substantial between-ward variance even when adjusting for patients’ individual psychopathology.

Regarding the use of coercive measures in different countries, Kalisova et al. [35] found a higher frequency of the use of coercive measures in Poland, Italy and Greece compared to the other seven analyzed European countries.

### Aims of study

Following up to these investigations, it was the aim of the present study to examine which role clinical and sociodemographic characteristics play regarding the admission to a locked ward and the application of coercive measures in comparison to the role of the clinic a patient is admitted to. We wanted to test our hypothesis that, in addition to the effect of patient characteristics on the admission to a locked ward and the use of coercive measures, there is also a clinic-related effect on the application of these measures.

## Methods

### General framework

The “Dokumentationsverbund Psychiatrie” (DVP) was established in 1984 to encourage benchmarking, quality management, and quality assurance in psychiatric hospitals in North Rhine-Westphalia, Lower Saxony, and Hesse (Germany) [36]. All 22 participating hospitals provide psychiatric and physical health care, offer community mental health services, are legally obliged to provide health care to all individuals from a specific area, and are part of a single-tier psychiatric care system. They are situated within larger general hospital complexes.

### Study population

We received informed consent from the medical director or holder of the equivalent position of 21 (95%) of the 22 hospitals actively participating in the DVP to use their data for analyses. 14 of the 21 hospitals had locked and open wards during the whole study period. Inclusion criteria were treatment in one of these 14 hospitals within the period from Jan 1, 1998, to Dec 31, 2012. Hospital stays before 1998, or with discharge after 2012, were excluded. We defined no further inclusion or exclusion criteria to ensure a naturalistic sample. The resulting dataset contained 230,684 hospital admissions of 177,295 individual patients.

### Documentation and management of clinical data

Data were recorded as part of routine treatment and all data that potentially allow the identification of individual patients were anonymized before the authors received the data for analysis [15, 16]. Therefore, no specific approval from the local ethics committee was required. The study was performed in accordance with all national and international legal regulations, and with the 7th revision of the Declaration of Helsinki (2013). Hospitals participating in the DVP collect structured routine data with a questionnaire that is the identical and used in the same way across hospitals. The survey includes a set of instructions to ensure correct assessments and the postal and email addresses and phone numbers of the main office to simplify direct enquiries. Furthermore, there is a separate documentation guide. Paper and electronic surveys are continuously completed by the treating physician and are then sent to the central office, where data entry, coding, cleaning, and plausibility checks are done.

We used a binary dependent variable to classify the type of admission as admission to a locked versus admission to an open ward. As a second binary dependent variable we used the application of coercive measures (fixation and/or seclusion).

### Statistical analysis

Descriptive statistics are given in total numbers and percentages for nominal scaled variables as well as mean and standard deviation (SD) for ordinal and interval scaled variables. Group comparisons were performed via chi-square tests (nominal scale, parametric) and one-way ANOVAs (ordinal and interval scale, parametric). Due to the descriptive nature of the exploratory comparisons accompanying and enhancing the sample description (Tables 1 and 2), no correction for multiple testing was employed.

**Table 1:** Clinical and sociodemographic characteristics for cases with admission to locked and open wards (*N* = 230’684)

	Locked Ward	Open Ward	*p*-value
**Number of cases**	100,979	129,705	
**Age (years)**	46.6 ± 17.6	48.0 ± 16.9	*p* < .001^a^
**Gender**			
Female	44,370 (43.9%)	68,837 (53.1%)	*p* < .001^b^
Male	56,562 (56.0%)	60,816 (46.9%)	*p* < .001^b^
Unknown	47 (< 0.1%)	52 (< 0.1%)	*p* = .261^b^
**German nationality**	95,890 (95.0%)	124,431 (95.9%)	*p* < .001^b^
**Marital status**			
Unmarried	44,321 (43.9%)	45,184 (34.8%)	*p* = .001^b^
Married	23,821 (23.6%)	42,361 (32.7%)	*p* = .001^b^
Separated/divorced	29,259 (29.0%)	38,950 (30.0%)	*p* = .001^b^
Unknown	3578 (3.5%)	3210 (2.5%)	*p* < .001^b^
**Housing situation**			
Private residence	86,514 (85.7%)	119,578 (92.2%)	*p* = .001^b^
Assisted living	12,007 (11.9%)	8451 (6.5%)	*p* = .001^b^
Homeless	1710 (1.7%)	1214 (0.9%)	*p* = .001^b^
Unknown	748 (0.7%)	462 (0.4%)	*p* = .001^b^
**Occupational situation**			
Employed	16,679 (16.5%)	30,796 (23.7%)	*p* < .001^b^
Education	3669 (3.6%)	5657 (4.4%)	*p* < .001^b^
Retired	29,909 (29.6%)	39,452 (30.4%)	*p* < .001^b^
Unemployed	33,547 (33.2%)	34,468 (26.6%)	*p* < .001^b^
Other types	9971 (9.9%	14,084 (10.9%)	*p* < .001^b^
Unknown	7204 (7.1%)	5248 (4.0%)	*p* < .001^b^
**Main diagnosis (ICD-10)**			
F0 Organic, including symptomatic, mental disorders	10,245 (10.1%)	7234 (5.6%)	*p* < .001^b^
F1 Mental and behavioral disorders due to psychoactive substance use	32,202 (31.9%)	32,408 (25.0%)	*p* < .001^b^
F2 Schizophrenia, schizotypal and delusional disorders	25,598 (25.3%)	19,854 (15.3%)	*p* < .001^b^
F3 Mood (affective) disorders	16,234 (16.1%)	46,960 (36.2%)	*p* < .001^b^
F4 Neurotic, stress-related and somatoform disorders	11,742 (11.6%)	16,542 (12.8%)	*p* < .001^b^
F6 Disorders of adult personality and behavior	4677 (4.6%)	5949 (4.6%)	*p* = .308^b^
Other psychiatric diagnosis	147 (0.1%)	666 (0.5%)	*p* = .678^b^
Unknown	134 (0.1%)	92 (0.1%)	*p* < .001^b^
**Comorbidities**			
Comorbidity with F1	45,450 (45.0%)	47,071 (36.3%)	*p* < .001^b^
Comorbidity with F6	11,201 (11.1%)	18,958 (14.6%)	*p* < .001^b^
Comorbidity with F7	2198 (2.2%)	1219 (0.9%)	*p* < .001^b^
**Voluntary entry**	76,025 (75.3%)	125,855 (97.0%)	*p* < .001^b^
**Type of admission**			
Patient’s initiative	35,958 (35.6%)	75,255 (58.0%)	*p* < .001^b^
Admission by physician	20,135 (19.9%)	25,421 (19.6%)	*p* = .021^b^
Compulsory admission	10,042 (9.9%)	2109 (1.6%)	*p* < .001^b^
Other types of admission	31,515 (31.2%)	23,790 (18.3%)	*p* < .001^b^
Unknown	3329 (3.3%)	3130 (2.4%)	*p* < .001^b^
**Behavior before admission**			
Aggressive behavior	14,687 (14.5%)	6028 (4.6%)	*p* < .001^b^
Suicide attempt	8910 (8.8%)	5026 (3.9%)	*p* < .001^b^
Suicidality	24,316 (24.1%)	24,204 (18.7%)	*p* < .001^b^
Unknown	744 (0.7%)	610 (0.5%)	*p* < .001^b^
**Clinic affiliation**			
Clinic 1	4908 (4.9%)	21,708 (16.7%)	*p* < .001^b^
Clinic 2	4567 (4.5%)	9055 (7.0%)	*p* < .001^b^
Clinic 3	5436 (5.4%)	13,179 (10.2%)	*p* < .001^b^
Clinic 4	1193 (1.2%)	7014 (5.4%)	*p* < .001^b^
Clinic 5	15,177 (15.0%)	2211 (1.7%)	*p* < .001^b^
Clinic 6	6297 (6.2%)	12,601 (9.7%)	*p* < .001^b^
Clinic 7	3469 (3.4%)	4523 (3.5%)	*p* = .254^b^
Clinic 8	2363 (2.3%)	5793 (4.5%)	*p* < .001^b^
Clinic 9	3641 (3.6%)	2882 (2.2%)	*p* < .001^b^
Clinic 10	12,159 (12.0%)	9754 (7.5%)	*p* < .001^b^
Clinic 11	12,916 (12.8%)	6820 (5.3%)	*p* < .001^b^
Clinic 12	12,424 (12.3%)	10,421 (8.0%)	*p* < .001^b^
Clinic 13	4327 (4.3%)	6226 (4.8%)	*p* < .001^b^
Clinic 14	5675 (5.6%)	14,376 (11.1%)	*p* < .001^b^

Values are given as number (percentage) for nominal variables and in mean ± standard deviation for continuous variables. To enhance the interpretability of the sample description, *p*-values from exploratory analyses are presented. ^a^: One-way ANOVA, ^b^: χ^2^-test

**Table 2:** Clinical and sociodemographic characteristics for cases with and without coercive measures during inpatient treatment (*N* = 230’684)

	Coercive measure	No coercive measure	*p*-value
**Number of cases**	15,730	214,954	
**Age (years)**	51.4 ± 19.9	47.1 ± 17.0	*p* < .001^a^
**Gender**			
Female	5807 (36.9%)	107,400 (50.0%)	*p* < .001^b^
Male	9916 (63.0%)	107,462 (50.0%)	*p* < .001^b^
Unknown	7 (< 0.1%)	92 (< 0.1%)	*p* = .517^b^
**German nationality**	14,895 (94.7%)	205,426 (95.6%)	*p* < .001^b^
**Marital status**			
Unmarried	6855 (43.6%)	82,650 (38.5%)	*p* = .001^b^
Married	3602 (22.9%)	62,580 (29.1%)	*p* = .001^b^
Separated/divorced	4610 (29.3%)	63,599 (29.6%)	*p* = .231^b^
Unknown	663 (4.2%)	6125 (2.8%)	*p* < .001^b^
**Housing situation**			
Private residence	12,517 (79.6%)	193,575 (90.1%)	*p* = .001^b^
Assisted living	2722 (17.3%)	17,736 (8.3%)	*p* = .001^b^
Homeless	344 (2.2%)	2580 (1.2%)	*p* = .001^b^
Unknown	147 (0.9%)	1063 (0.5%)	*p* = .001^b^
**Occupational situation**			
Employed	1646 (10.5%)	45,829 (21.3%)	*p* < .001^b^
Education	362 (2.3%)	8964 (4.2%)	*p* < .001^b^
Retired	6337 (40.3%)	63,024 (29.3%)	*p* < .001^b^
Unemployed	5096 (32.4%)	62,919 (23.3%)	*p* < .001^b^
Other types	1124 (7.1%)	22931 (10.7%	*p* < .001^b^
Unknown	1165 (7.4%)	11,287 (5.3%)	*p* < .001^b^
**Main diagnosis (ICD-10)**			
F0 Organic, including symptomatic, mental disorders	3578 (22.7%)	13,901 (6.5%)	*p* < .001^b^
F1 Mental and behavioral disorders due to psychoactive substance use	5391 (34.3%)	59,219 (27.5%)	*p* < .001^b^
F2 Schizophrenia, schizotypal and delusional disorders	4247 (27.0%)	41,205 (19.2%)	*p* < .001^b^
F3 Mood (affective) disorders	989 (6.3%)	62,205 (28.9%)	*p* < .001^b^
F4 Neurotic, stress-related and somatoform disorders	784 (5.0%)	27,500 (12.8%)	*p* < .001^b^
F6 Disorders of adult personality and behavior	691 (4.4%)	9935 (4.6%)	*p* = .096^b^
Other psychiatric diagnosis	27 (0.2%)	786 (0.4%)	*p* < .001^b^
>Unknown	23 (0.1%)	203 (0.1%)	*p* = .031^b^
**Comorbidities**			
Comorbidity with F1	7030 (44.7%)	85,491 (39.8%)	*p* < .001^b^
Comorbidity with F6	1406 (8.9%)	28,753 (11.4%)	*p* < .001^b^
Comorbidity with F7	395 (2.5%)	3022 (1.4%)	*p* < .001^b^
**Voluntary entry**	5113 (32.5%)	196,767 (91.5%)	*p* < .001^b^
**Type of admission**			
Patient’s initiative	1995 (12.7%)	109,218 (50.8%)	*p* < .001^b^
Admission by physician	3408 (21.7%)	42,848 (19.6%)	*p* < .001^b^
Compulsory admission	3341 (21.2%)	8810 (4.1%)	*p* < .001^b^
Other types of admission	6536 (41.6%)	48,769 (22.7%)	*p* < .001^b^
Unknown	450 (2.9%)	6009 (2.8%)	*p* = .325^b^
**Behavior before admission**			
Aggressive behavior	4912 (31.2%)	15,803 (7.4%)	*p* < .001^b^
Suicide attempt	732 (4.7%)	13,204 (6.1%)	*p* < .001^b^
Suicidality	2509 (16.0%)	46,011 (21.4%)	*p* < .001^b^
Unknown	131 (0.8%)	1223 (0.6%)	*p* < .001^b^
**Clinic affiliation**			
Clinic 1	500 (3.2%)	26,116 (12.1%)	*p* < .001^b^
Clinic 2	661 (4.2%)	12,961 (6.0%)	*p* < .001^b^
Clinic 3	1410 (9.0%)	17,205 (8.0%)	*p* < .001^b^
Clinic 4	72 (0.5%)	8135 (3.8%)	*p* < .001^b^
Clinic 5	851 (5.4%)	16537 (7.7%	*p* < .001^b^
Clinic 6	1327 (8.4%)	17,571 (8.2%)	*p* = .127^b^
Clinic 7	561 (3.6%)	7431 (3.5%)	*p* = .241^b^
Clinic 8	625 (4.0%)	7531 (3.5%)	*p* = .001^b^
Clinic 9	514 (3.3%)	6009 (2.8%)	*p* < .001^b^
Clinic 10	1287 (8.2%)	20,626 (9.6%)	*p* < .001^b^
Clinic 11	1613 (10.3%)	18,123 (8.4%)	*p* < .001^b^
Clinic 12	4653 (29.6%)	18,192 (8.5%)	*p* < .001^b^
Clinic 13	300 (1.9%)	10,253 (4.8%)	*p* < .001^b^
Clinic 14	724 (4.6%)	19,327 (9.0%)	*p* < .001^b^

Values are given as number (percentage) for nominal variables and in mean ± standard deviation for continuous variables. To enhance the interpretability of the sample description, *p*-values from exploratory analyses are presented. ^a^: One-way ANOVA, ^b^: χ^2^-test

To investigate the association of type of admission and the application of coercive measures with sociodemographic and clinical characteristics and the admission to a certain clinic, we performed a two-step multivariate logistic regression with the binary dependent variable “type of admission” (open vs. closed) and “application of coercive measures” (yes vs. no) and the variables listed in Tables 1 and 2 as independent variables.

To compare the increase of explained variance by including the different clinics as independent variables into the logistic regression model regarding type of admission and application of coercive measures, we calculated Nagelkerke’s R^2^ for each step of the logistic regression analysis.

All tests of significance were 2-tailed, and *p*-values < 0.05 were considered significant. Referring to Chen [37], we interpreted the odds ratios as follows: OR = 1.68, 3.47, and 6.71 are considered equivalent to Cohen’s d = 0.2 (small), 0.5 (medium), and 0.8 (large), respectively. Statistical analyses were conducted using PASW Statistics 18.0 (Chicago, Illinois, USA).

## Results

230,684 cases were admitted during the observation period. 129’705 cases were admitted to an open ward and 100’979 cases to a closed ward. The treatment of 15’730 cases included coercive measures, 214’954 were treated without coercive measures. Tables 1 and 2 show the clinical and sociodemographic characteristics of the cases at admission.

Patients admitted to locked wards (*M* = 46.6, *SD* = 17.6 years) were younger than patients admitted to open wards (*M* = 48.0, *SD* = 16.9 years). More patients admitted to locked wards (56.0%) were male than patients admitted to open wards (46.9%). Most patients were unmarried in both groups, with a higher percentage in patients admitted to locked wards (43.9% vs. 34.8%). Assisted living was more frequent in the group of subjects admitted to a locked ward (11.9% vs. 6.5%). Concerning the occupational situation, there was a lower percentage of employed subjects in the group of subjects admitted to a locked ward (16.5%) than in the group of patients admitted to an open ward (23.7%). Organic, including symptomatic, mental disorders (10.1% vs. 5.6%), mental and behavioral disorders due to psychoactive substance use (31.9% vs. 25.0%), and schizophrenia, schizotypal and delusional disorders (25.3% vs. 15.3%) were more frequent in the group of persons admitted to locked wards, whereas mood disorders (16.1% vs. 36.2%), neurotic, stress-related and somatoform disorders (11.6% vs. 12.8%) were more frequent in the group of subjects admitted to open wards. Comorbidities with mental and behavioral disorders due to psychoactive substance use (45.0% vs. 36.3%) or intellectual disabilities (2.2% vs. 0.9%) were more frequent among subjects admitted to locked wards. Voluntary entries were less frequent among persons admitted to locked wards (75.3% vs. 97.0%), as were admissions on a patient’s initiative (35.6% vs. 58.0%). Compulsory admissions (9.9% vs. 1.6%) were more frequent among patients admitted to locked wards. There were more persons showing aggressive behavior (14.5% vs. 4.6%), suicide attempts (8.8% vs. 3.9%) and suicidality (24.1% vs. 18.7%) before treatment in the group of persons admitted to a locked ward. The distribution of admissions to a locked vs. admission to an open ward varied across the participating clinics, with, e.g., Clinic 5 contributing 15.0% of admissions to locked wards and 1.7% of admissions to open wards, compared to Clinic 1 contributing 4.9% and 16.7%, respectively.

Patients whose treatment included coercive measures (*M* = 51.4, *SD* = 19.9) were older than patients whose treatment included no coercive measures (*M* = 47.1, *SD* = 17.0). Treatment including coercive measures was more frequent concerning male (63.0%) compared to female persons (36.9%). Among patients whose treatment included coercive measures, there were more unmarried (43.6%) and less married (22.9%) subjects than among patients whose treatment included no coercive measures (38.5% and 29.1%, respectively). Assisted living (17.3% vs. 8.3%) and homelessness (2.2% vs. 1.2%) were more frequent in the group of patients whose treatment included coercive measures. Concerning the occupational situation, there was a smaller percentage of employed subjects in the group of subjects whose treatment included coercive measures than in the group of subjects whose treatment included no coercive measures (10.5% vs. 21.3%). Retired and unemployed subjects were more frequent in the group of subjects whose treatment included coercive measures (40.3% vs. 29.3% and 32.4% vs. 23.3%, respectively). Organic, including symptomatic, mental disorders (22.7% vs. 6.5%), mental and behavioral disorders due to psychoactive substance use (34.3% vs. 27.5%), and schizophrenia, schizotypal and delusional disorders (27.0% vs. 19.2%) were more frequent in the group of subjects whose treatment included coercive measures, whereas mood disorders (6.3% vs. 28.9%), and neurotic, stress-related and somatoform disorders (5.0% vs. 12.8%) were more frequent in the group of subjects whose treatment included no coercive measures. Comorbidities with mental and behavioral disorders due to psychoactive substance use (44.7% vs. 39.8%) or intellectual disabilities (2.5% vs. 1.4%) were more frequent among subjects whose treatment included coercive measures. Voluntary entries were less frequent among subjects whose treatment included coercive measures (32.5% vs. 91.5%), as was admission on patient’s initiative (12.7% vs. 50.8%). Compulsory admissions (21.2% vs. 4.1%) and admissions by a physician (21.7% vs. 19.6%) were more frequent among subjects whose treatment included coercive measures. There were more subjects showing aggressive behavior before treatment (31.2% vs. 7.4%) among subjects whose treatment included coercive measures, whereas suicide attempts (4.7% vs. 6.1%) and suicidality (16.0% vs. 21.4%) before treatment were less frequent. Again, the distribution of coercive measures across the participating clinics varied considerably, with, e.g., Clinic 12 contributing 29.6% of cases with coercive measures and 8.5% of cases without coercion, compared to Clinic 1 contributing 3.2% and 12.1%, respectively.

Table 3 shows the final model of the logistic regression analysis for the dependent variable “admission to locked ward”. The best predictor with large effect size was an organic, including symptomatic, mental disorder (OR-1 = 7.454). Additionally, the associations between admission to a locked ward and the following variables indicated medium to large effect sizes: Clinic 1 (OR-1 = 6.692), schizophrenia, schizotypal and delusional disorders (OR-1 = 6.677), voluntary entry (OR-1 = 5.369), disorders of adult personality and behavior (OR-1 = 4.990), clinic 4 (OR-1 = 4.988) and 8 (OR = 4.848), mental and behavioral disorders due to psychoactive substance use (OR-1 = 4.679), clinic 5 (OR-1 = 4.285) and 3 (OR-1 = 3.525) and neurotic, stress-related and somatoform disorders (OR-1 = 3.472).

**Table 3:** Logistic regression analysis for the dependent variable “admission to locked ward”

Admission to locked ward	B	SE	df	*p*	Exp(B)	95% CI	Exp(B) abs − 1
F0 Organic, including symptomatic, mental disorders	2.135	0.101	1	< 0.001	8.454	6.939–10.300	7.454
Clinic 1	-2.04	0.031	1	< 0.001	0.13	0.122 − 0.138	6.692
F2 Schizophrenia. schizotypal and delusional disorders	2.038	0.098	1	< 0.001	7.677	6.329–9.311	6.677
Voluntary entry	-1.852	0.02	1	< 0.001	0.157	0.151 − 0.163	5.369
F6 Disorders of adult personality and behavior	1.79	0.102	1	< 0.001	5.99	4.907–7.312	4.990
Clinic 4	-1.793	0.043	1	< 0.001	0.167	0.153 − 0.181	4.988
Clinic 8	-1.766	0.039	1	< 0.001	0.171	0.158 − 0.185	4.848
F1 Mental and behavioral disorders due to psychoactive substance use	1.737	0.099	1	< 0.001	5.679	4.674–6.901	4.679
Clinic 5	1.665	0.035	1	< 0.001	5.285	4.931–5.665	4.285
Clinic 3	-1.508	0.032	1	< 0.001	0.221	0.208 − 0.235	3.525
F4 Neurotic. stress-related and somatoform disorders	1.498	0.099	1	< 0.001	4.472	3.686–5.427	3.472
Clinic 14	-1.468	0.031	1	< 0.001	0.23	0.217 − 0.245	3.348
Suicide attempt	1.408	0.039	1	< 0.001	4.088	3.740–4.468	3.088
Clinic 6	-1.293	0.031	1	< 0.001	0.275	0.259 − 0.292	2.636
Clinic 2	-1.2	0.032	1	< 0.001	0.301	0.283 − 0.321	2.322
F3 Mood (affective) disorders	1.045	0.099	1	< 0.001	2.843	2.343–3.449	1.843
Clinic 7	-0.863	0.037	1	< 0.001	0.422	0.393 − 0.453	1.370
Clinic 13	-0.757	0.034	1	< 0.001	0.469	0.439 − 0.501	1.132
Compulsory admission	0.728	0.03	1	< 0.001	2.071	1.954–2.195	1.071
Suicidality	0.722	0.017	1	< 0.001	2.059	1.990–2.130	1.059
Clinic 12	-0.669	0.03	1	< 0.001	0.512	0.483 − 0.543	0.953
Patient’s initiative	-0.613	0.013	1	< 0.001	0.542	0.528 − 0.556	0.845
Aggressive Behavior	0.452	0.02	1	< 0.001	1.572	1.512–1.634	0.572
Admission by physician	-0.38	0.016	1	< 0.001	0.684	0.663 − 0.705	0.462
Comorbidity with F7	0.325	0.044	1	< 0.001	1.384	1.268–1.510	0.384
Comorbidity with F1	0.321	0.017	1	< 0.001	1.379	1.334–1.425	0.379
Education	-0.296	0.032	1	< 0.001	0.743	0.698 − 0.792	0.346
Clinic 9	-0.28	0.039	1	< 0.001	0.756	0.701 − 0.816	0.323
Private residence	-0.265	0.047	1	< 0.001	0.767	0.699 − 0.842	0.304
Clinic 10	-0.243	0.03	1	< 0.001	0.784	0.740 − 0.831	0.276
Comorbidity with F6	-0.217	0.02	1	< 0.001	0.805	0.774 − 0.838	0.242
Employed	-0.196	0.02	1	< 0.001	0.822	0.790 − 0.855	0.217
Unmarried	0.152	0.024	1	< 0.001	1.164	1.111–1.220	0.164
Married	-0.12	0.022	1	< 0.001	0.887	0.850 − 0.926	0.127
German nationality	-0.097	0.025	1	< 0.001	0.907	0.864 − 0.952	0.103
Sex (male)	0.085	0.011	1	< 0.001	1.088	1.066–1.112	0.088
Unemployed	0.083	0.021	1	< 0.001	1.087	1.043–1.133	0.087
Assisted living	-0.059	0.05	1	0.242	0.943	0.855 − 1.040	0.060
Clinic 11	0.059	0.03	1	0.054	1.06	0.999–1.125	0.060
Retired	0.023	0.022	1	0.293	1.023	0.980–1.068	0.023
Year of admission	0.018	0.001	1	< 0.001	1.018	1.015–1.020	0.018
Age	-0.008	0.001	1	< 0.001	0.993	0.992 − 0.994	0.007
Separated	0.007	0.023	1	0.763	1.007	0.962–1.054	0.007

Regarding the admission to a locked ward, clinical and sociodemographic variables explained 26.9% of the variance (Nagelkerke’s R^2^ = 0.269). By the addition of the different clinics into the logistic regression model as independent variables, 14.9% more of the variance could be explained (Nagelkerke’s R^2^ = 0.418).

Table 4 shows the final model of the logistic regression analysis for the dependent variable “coercive measures”. The best predictor of the application of coercive measures was voluntary entry (OR-1 = 8.805). Being admitted to clinic 12 had a medium effect size association (OR-1 = 3.656) with the application of coercive measures.

**Table 4:** Logistic regression analysis for the dependent variable “coercive measures”

Coercive measures	B	SE	df	*p*	Exp(B)	95% CI	Exp(B) abs − 1
Voluntary entry	-2.284	0.022	1	< 0.001	0.102	0.098 − 0.106	8.804
Clinic 12	1.538	0.053	1	< 0.001	4.656	4.198–5.165	3.656
F0 Organic, including symptomatic, mental disorders	1.187	0.23	1	< 0.001	3.278	2.090–5.142	2.278
Patient’s initiative	-0.811	0.03	1	< 0.001	0.445	0.419 − 0.472	1.247
Clinic 4	-0.786	0.134	1	< 0.001	0.456	0.351 − 0.592	1.193
Clinic 6	0.662	0.058	1	< 0.001	1.939	1.729–2.174	0.939
Clinic 3	0.648	0.058	1	< 0.001	1.911	1.705–2.143	0.911
Aggressive Behavior	0.639	0.024	1	< 0.001	1.895	1.810–1.985	0.895
F1 Mental and behavioral disorders due to psychoactive substance use	0.631	0.23	1	0.006	1.88	1.198–2.949	0.880
F6 Disorders of adult personality and behavior	0.596	0.235	1	0.011	1.814	1.145–2.876	0.814
Clinic 9	0.566	0.073	1	< 0.001	1.762	1.526–2.034	0.762
Clinic 13	-0.524	0.08	1	< 0.001	0.592	0.506 − 0.693	0.689
Clinic 8	0.522	0.068	1	< 0.001	1.685	1.475–1.925	0.685
Clinic 1	-0.416	0.068	1	< 0.001	0.659	0.577 − 0.754	0.517
F3 Mood (affective) disorders	-0.391	0.23	1	0.089	0.676	0.430–1.062	0.479
F2 Schizophrenia. schizotypal and delusional disorders	0.386	0.228	1	0.09	1.471	0.941 − 2.300	0.471
Clinic 11	0.374	0.057	1	0.054	1.453	1.300–1.625	0.453
Clinic 2	0.361	0.066	1	< 0.001	1.435	1.262–1.632	0.435
Comorbidity with F7	0.356	0.068	1	< 0.001	1.428	1.249–1.633	0.428
Compulsory admission	0.338	0.031	1	< 0.001	1.402	1.320–1.489	0.402
Assisted living	0.287	0.079	1	0.242	1.332	1.142–1.554	0.332
Clinic 7	0.271	0.071	1	< 0.001	1.311	1.141–1.506	0.311
F4 Neurotic. stress-related and somatoform disorders	-0.262	0.231	1	0.256	0.77	0.490 − 1.210	0.299
Clinic 14	-0.245	0.064	1	< 0.001	0.783	0.691 − 0.887	0.277
Sex (male)	0.201	0.019	1	< 0.001	1.223	1.177–1.270	0.223
Married	0.188	0.041	1	< 0.001	1.207	1.114–1.307	0.207
Admission by physician	-0.183	0.026	1	< 0.001	0.833	0.791 − 0.877	0.200
Clinic 5	0.176	0.063	1	0.006	1.192	1.053–1.350	0.192
Retired	0.155	0.043	1	0.293	1.167	1.073–1.270	0.167
Comorbidity with F6	0.146	0.046	1	0.002	1.158	1.057–1.268	0.158
Unmarried	0.144	0.043	1	0.001	1.155	1.062–1.256	0.155
Suicidality	-0.138	0.027	1	< 0.001	0.871	0.826 − 0.919	0.148
Employed	-0.126	0.046	1	0.007	0.882	0.805 − 0.966	0.134
Clinic 10	0.119	0.058	1	0.042	1.126	1.004–1.262	0.126
Private residence	-0.095	0.074	1	0.2	0.909	0.786–1.052	0.100
Unemployed	0.044	0.041	1	0.285	1.045	0.964–1.132	0.045
Year of admission	-0.036	0.002	1	< 0.001	0.965	0.960 − 0.970	0.036
Education	-0.026	0.074	1	0.726	0.974	0.841–1.128	0.027
Comorbidity with F1	0.023	0.034	1	0.487	1.024	0.958–1.094	0.024
Suicide attempt	-0.015	0.048	1	0.757	0.985	0.896–1.084	0.015
German nationality	0.009	0.046	1	0.839	1.009	0.922–1.104	0.009
Separated	0.006	0.042	1	0.894	1.006	0.927–1.091	0.006
Age	0	0.001	1	0.956	1	0.998–1.002	0.000

Regarding the application of coercive measures, clinical and sociodemographic variables explained 36.4% of the variance (Nagelkerke’s R^2^ = 0.364). By the addition of the different clinics into the logistic regression model as independent variables, 3.6% more of the variance could be explained (Nagelkerke’s R^2^ = 0.400).

## Discussion

The present 15-year, naturalistic, observational study examined which role sociodemographic and clinical characteristics play regarding the admission to a locked ward and the application of coercive measures in comparison to the role of being admitted to a specific clinic. To the authors’ knowledge, this is the first study comparing the predictive value of patient- and clinic-related characteristics concerning the admission to locked wards and coercive measures.

The differences we found concerning sociodemographic characteristics of our sample are in line with previous research findings [16, 38, 39]. We established that in terms of diagnosis group, organic, including symptomatic, mental disorders were the diagnoses most strongly associated with coercive measures and admission to a locked ward. A possible explanation is the high percentage of people with dementia showing aggressive behavior during their course of illness [40, 41]. Involuntary entry were moderately to strongly associated with the admission to a locked ward. Regarding coercive measures, involuntary entry was the most important predictor of using coercive measures during treatment. The clinical predictors of the admission to a locked ward and of coercive measures are in line with previous literature [42, 43]. When we compare the independent variables “admission to a locked ward” and “coercive measures” it is noticeable that the decision to place patients in a locked ward is more strongly influenced by patient characteristics than the application of coercive measures. A possible explanation for these differences could lie in the treatment course: The decision that a patient has to be admitted to a locked ward is often made in the very beginning of the inpatient treatment. Therefore, anamnestic data is of important relevance for this decision. On the other hand, coercive measures are often used in the course of treatment, which places the clinical assessment at the center.

In addition to the patient-related predictors, being treated in a specific clinic also predicted admission to a locked ward and coercion. Probability of the admission to a locked ward was moderately elevated for one clinic and moderately or strongly lowered for four clinics. Besides, there was one clinic, where the probability of using coercive measures was moderately elevated. The predictive value of admission to a specific clinic was higher regarding locked doors than coercive measures, whereas the predictive value of patient-related factors was higher regarding coercive measures than locked doors. This in line with, e.g., the results of the EUNOMIA research group [35] and further research concerning the varying use of coercion in psychiatry [44–47].

In summary, these findings are compatible with previous literature showing that patient-related factors and local tradition both predict the use of coercive measures in psychiatry [32]. They suggest that local tradition plays a role for both admission to a locked ward and coercive measures, and that the role of local tradition is more prominent regarding admission to a locked ward. However, taking into account that the investigated variables could only explain 41.8% (door status closed at admission) and 40.0% (application of coercive measures) of the variance, respectively, we must assume that there are further important predictors, which we could not examine in our study.

### Strengths

Important strengths of our study are the naturalistic study design and the large sample size and long-term observation period, enhancing the generalizability of our findings. Furthermore, a potential admission bias was reduced by the fact that all clinics included in the study were obliged to admit all patients from their catchment area. Additionally, the quality of the collected routine data was enhanced by the inclusion of instructions and contact data on questionnaires, a documentation guide and centralized data management. The diversity of the included hospitals concerning ownership (government, non-profit, private health-care provider) and catchment area (rural vs. urban) enhance the generalizability of the results and ensure an apt representation of the German health care system.

### Limitations

Besides several strengths of our study, there are also limitations. Whereas the large sample size including patients with the whole spectrum of psychiatric diagnoses, the inclusion of hospitals from different catchment areas and the minimal in- and exclusion criteria enhance the generalizability of our findings, generalizability is limited by the fact that we examined only German clinics. It is unclear, to what extent our results are transferable to and helpful for other countries with other health care systems.

Furthermore, local differences concerning the use of coercive measures and admissions to locked wards could in theory be the effect of differences in treated patients, e.g., regarding diagnostic composition or aggression and suicidality at admission. However, the presented analyses controlled for several key patient-related variables related to coercion and treatment on locked wards in the logistic regression analyses, and thus such biases are not likely. Future research, e.g., using a randomized controlled trial (RCT) design, is recommended to corroborate these findings.

Moreover, the current analyses were based on routine data containing further methodological limitations. Although they were prospectively entered in an electronic documentation system, and it is known that data quality and completeness is sufficient for scientific analyses [48], only basic clinical data were available. Other variables, e.g. forensic history or aggressive incidents during treatment, would have been of additional use.

Another consequence of analyzing routine data is the pre-established timeframe of data collection. We did not have the opportunity to analyze data from after 2012. Nevertheless, the research about this topic is still very scarce, which is why it is important to not only shed light on the current time period, but also to include earlier data to provide a more comprehensive understanding of the subject.

Lastly, local tradition is a very complex construct containing many different aspects (e.g. clinical tradition, structural requirements, legal practice, influence of local stakeholders) that we could not differentiate in our study.

### Further studies

Further studies should investigate the factors behind the local tradition and examine, which differences between clinics lead to a different handling of coercion in psychiatric inpatient treatment. Furthermore, other countries should be involved in future analyses to investigate, if the impact of local tradition differs between cultures. An important aspect of coercion in psychiatry is the informal coercion in the therapeutic relationship. Further studies should therefore include the whole spectrum of coercive measures in the sense of a continuum to ensure a more comprehensive view of the prediction of coercive measures. This could also play a role in the early prevention of coercion in psychiatry. Despite the fact that coercion is more frequently used in inpatient settings, future research investigating coercive measures in outpatient settings would also be important.

## Conclusion

The local tradition of the psychiatric clinic seems to play a more substantial role regarding the decision to admit a person to a locked ward than regarding the decision to use coercive measures. Hence, clinicians should be made aware to this potential bias to avoid unreasonable admissions to locked wards.

## Data Availability

Due to the nature of the research and the associated ethical restrictions data is not available.
